# Molecular Subtype Discordance in a Young Woman with Synchronous Bilateral Breast Cancer: A Case Report

**DOI:** 10.7759/cureus.7242

**Published:** 2020-03-11

**Authors:** Alejandro Aranda-Gutierrez, Analy Gomez-Picos, Ana S Ferrigno, Mariana Moncada-Madrazo, Hector Diaz-Perez

**Affiliations:** 1 Internal Medicine, Tecnologico de Monterrey, Escuela de Medicina y Ciencias de la Salud, Monterrey, MEX; 2 Tecnologico de Monterrey, Escuela de Medicina y Ciencias de la Salud, Monterrey, MEX; 3 Clinical Sciences, Tecnologico de Monterrey, Escuela de Medicina y Ciencias de la Salud, Monterrey, MEX; 4 Oncology, Tecnologico de Monterrey, Escuela de Medicina y Ciencias de la Salud, Monterrey, MEX

**Keywords:** breast cancer, bilateral breast cancer, synchronous neoplasm, discordant molecular subtype, hormone receptor

## Abstract

Molecular subtype discordance in bilateral synchronous breast cancer is a relatively uncommon entity that poses unique therapeutic challenges. Here we report the case of a 35-year-old woman who presented to our clinic with synchronous bilateral breast cancer (SBBC), with stage IIA triple-negative disease in the right breast and a stage IIB hormone-sensitive tumor in the left breast. She was treated with bilateral modified radical mastectomy and axillary node dissection followed by adjuvant chemotherapy, radiotherapy, and a five-year regimen with daily tamoxifen. To date, the patient remains asymptomatic and free of disease recurrence 78 months after initiating treatment. Little is known about SBBC with a discordant molecular subtype and reports about this entity are scarce. Future studies aimed at identifying the optimal management strategy for this disease are needed.

## Introduction

Synchronous bilateral breast cancer (SBBC) constitutes a therapeutic challenge about which scarce information is currently available. Although considered to be an uncommon disorder, its incidence is not negligible, as up to 7.5% of breast cancer patients have bilateral disease, with 38% of it classified as synchronous. Therefore, approximately 2.9% of all breast cancer cases can be classified as SBBC [1]. Nevertheless, its relatively low incidence coupled with the limited available scientific evidence has translated into a lack of guidelines regarding its optimal management.

Selecting an optimal treatment plan for patients with SBBC can be a daunting task for multiple reasons, including higher stage at presentation and increased mortality when compared to unilateral disease [2-3]. Additionally, this entity can be complicated by a phenomenon that is not significant in unilateral cases: inter-tumoral discordance. The latter can occur in histological type, grade of differentiation, molecular subtype, and/or clinical staging. Particularly, the presence of two synchronous tumors with differing molecular subtypes introduces notable complexity to the selection of a treatment strategy, as it impacts the expected therapeutic response, risk of recurrence, and prognosis [3-4]. The management of these cases becomes more complex when discordance occurs in hormone receptor status, which is estimated to affect 5% of SBBC cases [5]. 

The objective of this study is to report the case of a young woman diagnosed with SBBC with molecular subtype discordance, and review the relevant literature regarding this entity. 

## Case presentation

A 35-year-old premenopausal patient with class I obesity (defined as a body mass index between 30 and 34.9 kg/m2 ) and a family history of unilateral breast cancer in a sister and a paternal aunt underwent a routine mammogram, in which bilateral findings consistent with a moderate likelihood (10%-49%) for malignancy (BI-RADS 4B) were detected. Core-needle biopsies of the suspicious areas were performed, revealing the presence of invasive ductal carcinoma (IDC) in both breasts. Immunohistochemistry (IHQ) for estrogen receptor (ER), progesterone receptor (PR), and human epidermal growth factor receptor 2 (HER2) was performed on the tissue samples. Disease in the right breast was established as ER and PR negative with indeterminate HER2 status (subsequently confirmed as negative by fluorescence in situ hybridization). Disease in the left breast was ER and PR positive (with 60% and 50% expression, respectively), while HER2 expression was determined to be negative. Consequently, a diagnosis of SBBC with molecular subtype discordance was established. Staging studies were negative for distant metastatic disease. Thus, based on the seventh edition of the American Joint Committee on Cancer (AJCC) staging manual, clinical staging was established as follows: IIA (T2N0M0) for the right breast and IIB (T2N1M0) for the left. 

The patient underwent bilateral modified radical mastectomy with bilateral axillary lymph node dissection. The surgical pathology report indicated the following: 1) right breast: IDC, moderately differentiated, IHQ negative for ER, PR, and HER2, Ki-67 20%, tumor size of 2 cm, 17 axillary lymph nodes negative for metastasis, pT2N0M0; 2) left breast: IDC, poorly differentiated, positive for ER and PR and negative for HER2, Ki-67 30%, tumor size 4.5 cm, two of 17 axillary lymph nodes positive for metastasis, pT2N1M0. 

Adjuvant therapy with doxorubicin-cyclophosphamide (four cycles) followed by docetaxel (four cycles) was administered. Once adjuvant chemotherapy was completed, the patient underwent adjuvant radiotherapy with 50 Gy over 25 fractions. Because of the hormone-sensitive disease and premenopausal status, the patient was subsequently recommended hormone therapy to reduce disease recurrence risk, composed of tamoxifen 20 mg/day for a minimum of five years. To date, the patient has remained asymptomatic and without evidence of breast cancer recurrence 78 months after treatment initiation.

## Discussion

Various risk factors for bilateral breast cancer have been described, including young age, family history, BRCA1/2 gene mutation, lobular histologic type, and multicentricity. However, specific risk factors for developing molecular subtype discordance have not been established. Evidence demonstrates that the rate of discordance for ER in metachronous bilateral breast cancer is five times greater than it is in SBBC (25% vs. 5%) [5]. Thus, timing of diagnosis of each primary breast cancer could influence the associated risk factors, although further research is needed to elucidate the involved mechanisms. 

Synchronous bilateral breast cancer may present as two primary tumors or metastatic disease, even in the setting of molecular subtype discordance. The tumor origin could be further elucidated with gene sequencing techniques, as a strong correlation in single nucleotide variants indicates a shared clonal origin. Thus, bilateral disease with a high proportion of single nucleotide variants could be a marker for metastatic disease to the contralateral breast [6]. Nevertheless, the poor availability of exome sequencing tools, coupled with the uncertain clinical relevance of distinguishing between primary or metastatic origin, limits the utility of elucidating the origin of the tumors. Consequently, most clinicians treat SBBC as two separate, independent diseases.

Sighoko et al. propose that a significant proportion (53%-83%) of the discordance seen in ER in synchronous primary tumors represents a technical error during IHQ [5]. This represents a potential risk when indicating treatment, as patients could be exposed to the adverse effects of certain therapies (i.e. hormone therapy or anti-HER2 monoclonal antibodies) without receiving the expected benefit [7-8]. Independently of whether SBBC with molecular subtype discordance is primary or metastatic, or if discordance is due to technical errors, it has been reported that hormone receptor discordance is associated with increased rate of metastasis and higher mortality at five years of diagnosis [3, 9]. Thus, the indication of aggressive chemotherapy schemes appears to be justified. However, it is yet to be established if prognosis is solely dependent on the biology and stage of the most aggressive synchronous tumor.

It has been suggested that the election of systemic therapy for SBBC should be based on the prognosis of the most aggressive tumor, without clear supporting evidence [10]. Currently, available literature suggests that both chemotherapy and hormone therapy should be administered in the treatment of SBBC with hormone receptor discordance [3, 9, 11]. 

A literature search up to December 2019 was conducted for SBBC with molecular subtype discordance in the MEDLINE, EMBASE, and CENTRAL databases using the following search strategy in the title or abstract sections: “breast cancer” AND (“bilateral” OR “contralateral”) AND (“discordant” OR “discordance” OR “heterogeneous”). Articles in Spanish or English were considered to be eligible. Cross-referencing was performed to retrieve additional reports that could have been missed in the initial search. Given the scarcity of published reports, the search was not limited by date or publication. The initial search registered a total of 95 records, of which 20 were excluded for duplication, 28 for not corresponding to molecular subtype discordance, 20 for lacking case presentations, 8 for not corresponding to breast cancer, and 6 for corresponding to basic science topics. Only four previously published reports of SBBC with molecular subtype discordance were found (Table 1), illustrating the limited literature available for this entity [4, 9, 11-12]. The age at diagnosis in these reports was between 48 and 60 years, making our patient the youngest reported case to date. Discordance in hormone receptor status occurred in all cases, while discordance in HER2+ status occurred in only two cases [4, 12]. All cases received neoadjuvant chemotherapy, which was followed by bilateral modified radical mastectomy (MRM) in three patients [4, 11-12]. Regarding adjuvant therapy, two cases were initiated on hormone therapy for five years [4, 11]. Only one of them was accompanied by radiotherapy [11]. The remaining case initiated trastuzumab as monotherapy for nine months, subsequently adding letrozole for five years [12]. Long-term follow-up is reported for only two cases, with a patient remaining recurrence-free 20 months after diagnosis and another dying 71 months after starting treatment [11-12]. 

**Table 1:** Published case reports about SBBC with molecular subtype discordance. ALND, axillary lymph node dissection; HR, hormone receptor status; HER2, human epidermal growth factor receptor 2; MRM, modified radical mastectomy; SLN, sentinel lymph node; N/A, not available; SBBC, synchronous bilateral breast cancer *AJCC classification according to the authors

Reference	Age at diagnosis	Clinical stage*	IHQ	Treatment	Follow-up
Hayashi et al. (2013)	60 years	Right breast: IIIB (T4bN0M0) ; Left breast: IIIB (T4bN1M0)	Right breast: HR+/HER2- ; Left breast: HR-/HER2+	Neoadjuvant therapy (chemotherapy and trastuzumab), bilateral MRM with bilateral ALND, and adjuvant therapy (trastuzumab and letrozole)	The patient died after 71 months of follow-up, free of recurrence
Esclovon et al. (2016)	59 years	Right breast: N/A ; Left breast: N/A	Right breast: HR-/HER2+ ; Left breast: HR+/HER2-	Neoadjuvant therapy (chemotherapy and trastuzumab), bilateral MRM and right SLN, and adjuvant therapy (unspecified hormone therapy)	N/A
Copur et al. (2017)	48 years	Right breast: IIB (T2N1M0) ; Left breast: IIA (T2N0M0)	Right breast: HR-/HER2- ; Left breast: HR+/HER2-	Neoadjuvant therapy (chemotherapy), bilateral MRM with right ALND and left SNL, and adjuvant therapy (radiotherapy and unspecified hormone therapy)	20 months of follow-up, with no signs of recurrence
Dhadlie et al. (2018)	54 years	Right breast: N/A ; Left breast: N/A	Right breast: HR-/HER2+ ; Left breast: HR+/HER2+	Neoadjuvant therapy (chemotherapy and hormone therapy). Surgical and adjuvant management to be determined based on response to neoadjuvant therapy	N/A

The management of the young woman presented in this report was consistent with the available literature regarding SBBC with molecular subtype discordance [4, 9, 11-12]. The main difference was that, in our case, chemotherapy was administered in an adjuvant fashion. Even though the benefits of neoadjuvant chemotherapy are well established (it improves tumor resectability and aids in breast tissue preservation), there seems to be no significant long-term differences in clinical outcomes compared to the use of adjuvant chemotherapy [13]. In our case, we reported a follow-up of 78 months free of recurrence, supporting the effectiveness of the therapeutic interventions found in literature for this entity.

## Conclusions

Molecular subtype discordance introduces an important degree of complexity in the management of SBBC. In this report, we presented the case of a young woman with SBBC, with a triple negative tumor in the right breast and hormone-sensitive disease in the left breast. The patient was treated with bilateral MRM alongside systemic chemotherapy and hormone therapy and has remained recurrence-free for 78 months after starting treatment. To our knowledge, our study is the first case report of a young woman with SBBC with molecular subtype discordance.
